# The Treatment of Mammary Cancer

**Published:** 1898-04

**Authors:** 


### THE TREATMENT OF MAMMARY CANCER.

The prolonged discussion in one of the leading medical societies of England upon mammary cancer inevitably led to the conclusion that neither from the medical or surgical standpoint were we within a reasonable distance of acquiring a satisfactory method of dealing with this fell disease. A discussion, however, which defines the extent and limitations of knowledge is not entirely unavailing. The principal point raised by the discussion was whether the operative treatment of mammary cancer was really a treatment at all. If the statistics of the last ten years have any value, it seems doubtful if long immunity is in many cases due to operation. Any such term as the cure of cancer must generally be reprobated.

After surgical intervention, three years' freedom from recurrence has been accepted as a hopeful prognostic of immunity. Nothing, however, is surer than that such is not in any sense a guarantee of it. At best, the ordinary removal of the mamma leads to recoveries ranging from ten to eighteen per cent. Halstead's operation, which involves the removal of the glands of the axilla and the pectoral muscle, is averred to favor a recovery of forty per cent. This operation is, however, a formidable one, and it is scarcely proved that the ratio of improvement it secures offsets the increased mortality. It is also alleged with some show of reason that the greater operation favors a greater amount of visceral metastasis.

A rational therapeusis of cancer will undoubtedly never be achieved until clearer and more definite notions prevail as to its essential nature. There are some grounds for believing cancer to be at one time a local disease, and as such local measures might be expected to modify its course. Syphilis might as justly be regarded a local disease. Local measures, however, are incapable of counteracting its constitutional implantation. To remove a cancer may be as futile a proceeding as the removal of a chancre. If cancer be a local disease at any time, nothing is known of the period at which it becomes constitutional, and nothing of the process whereby it infects distant parts.

So indifferent is the success achieved by surgical intervention that it can only be regarded a failure even though it prove the most promising resort open to the desperate. It is encouraging to find activity manifested in other directions. From the therapeutical laboratory something is still to be expected. Promising indications have resulted from the formation of an experimental cirrhosis by means of dilute alcohol injections at the circumference of the tumor. Twenty-five cases of cure have already been reported. Formaldehyde also seems to exercise an extraordinary influence in these cases. The gleams of hope which occasionally irradiate this gloomiest domain of medicine should give encouragement to experimental activity in manifold directions.—*Medical Age.*

